# Adnexotropic Variants of the Interface Dermatitides: A Review

**DOI:** 10.3390/dermatopathology8020020

**Published:** 2021-05-21

**Authors:** Carla Stephan, Ossama Abbas, Jag Bhawan

**Affiliations:** 1 Department of Dermatology, American University of Beirut, Beirut 11-0236, Lebanon; cs53@aub.edu.lb (C.S.); ossamaabbas2003@yahoo.com (O.A.); 2 Department of Dermatology, Boston University School of Medicine, Boston, MA 02118-2415, USA

**Keywords:** interface dermatitis, folliculotropic, syringotropic, lichen planus, lichen sclerosus, graft versus host disease, lichen nitidus, lichen striatus, mycosis fungoides

## Abstract

The interface dermatitides encompass a vast array of cutaneous entities which, at times, may present with particular clinical variants with adnexal predilection. Similarly, hair follicle and eccrine gland involvement of some of these entities has been observed on histopathology. This review aims to describe the various adnexotropic presentations of the interface dermatitides. Recognizing that the adnexa can be a frequent site of involvement of these conditions may aid dermatopathologists in making the correct diagnosis and avoid misinterpreting adnexotropism for other conditions such as the great imitator, mycosis fungoides.

## 1. Introduction

Interface dermatitis is a pathological pattern characterized by the presence of basal cell vacuolization and apoptotic keratinocytes. A variety of dermatoses exhibit interface dermatitis on pathology including the lichenoid dermatoses, graft versus host disease, connective tissue diseases, and drug reactions, among others [1,2].

Several entities of interface dermatitides are known to have distinct rare adnexotropic variants whereby the inflammation involves the adnexa of the skin such as the hair follicle or the sweat gland. In lichen planus for example, follicular and syringotropic variants have been classically described.

Adnexal involvement can also at times be seen on histopathology of the interface dermatitides that do not have distinct adnexotropic variants. For example, adnexal inflammation can be seen in lichen striatus or in pityriasis lichenoides.

Recognizing the possibility of adnexal involvement in the interface dermatitides is important, as adnexal involvement is commonly seen in dermatopathological mimickers such as mycosis fungoides.

## 2. Interface Dermatitides with Distinct Clinicopathologic Adnexotropic Variants

### 2.1. Lichen Planus

**Figure 1:**
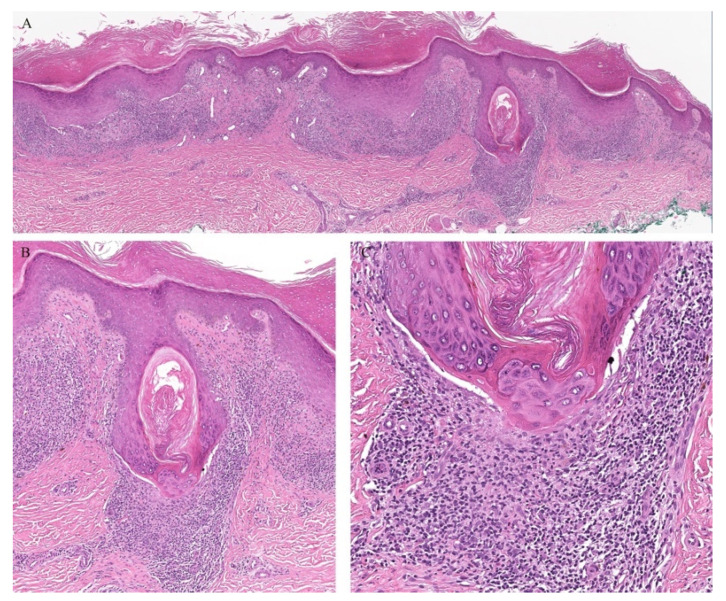
(**A**–**C**) Lichen planus with follicular involvement.

Lichen planus is the prototype lichenoid dermatitis characterized by a self-limiting eruption of pruritic flat-topped violaceous papules. On histopathology, hyperkeratosis, hypergranulosis, irregular acanthosis, and subepidermal clefts known as Max Joseph spaces are observed. A band-like lymphohistiocytic infiltrate is also seen. Distinct adnexotropic variants of lichen planus have been described (Figure 1). These include follicular lichen planus, lichen planus follicularis tumidus, and syringotropic lichen planus.

Lichen planopilaris, or follicular lichen planus, is a distinct variant of lichen planus whereby inflammation is seen around the hair follicle both clinically and histologically (Figure 1). Clinically, this presents with perifollicular erythema, follicular keratotic plugs, and a scarring alopecia [1,2].

Lichen planus follicularis tumidus (LPFT) is another distinct adnexotropic variant of lichen planus. Clinically, it is characterized by the presence of typical lichen planus lesions with the addition of comedo-like lesions and keratin-filled cysts, most commonly located in the retroauricular area. Most patients have classical lichen planus lesions on other parts of the body. On histopathology, the typical features of lichen planus are seen along with the involvement of the hair follicle. Follicular hyperkeratosis is observed and the follicular infundibulum is cystic and dilated. The follicular infundibulum is also surrounded by a lichenoid infiltrate [3]. Recognizing this entity is important as the differential diagnosis includes cutaneous lupus erythematosus (LE) with cysts and comedones and follicular mycosis fungoides (MF). Cutaneous LE is characterized by increased mucin deposition not seen in LPFT, while the presence of epidermotropism and an infiltrate of atypical lymphocytes is noted in follicular MF and not in LPFT [4].

Follicular involvement has also been rarely described in lichen planus pigmentosus (LPP). LPP is characterized by brown-gray macules over sun-exposed sites [1,2]. Histologically, this is reflected as lichenoid infiltrate with basal cell vacuolization and pigment incontinence. Although not generally considered as a folliculotropic variant of lichen planus, LPP has been described to involve the hair follicle. In a retrospective study of ninety-one patients with LPP, nine were found to have a perifollicular involvement. This included follicular plugging and a lymphohistiocytic perifollicular infiltrate: a histologic picture similar to that of lichen planopilaris. In addition, two of the nine patients had clinical features of lichen planopilaris on the scalp [5].

In addition to follicular variants of lichen planus, syringotropic variants have also been described. A classical eruption of lichen planus has been reported which on histopathology showed evidence of syringotropism. The term “acrosyringeal lichen planus” has been proposed by Enhamre and Lagerholm as a distinctive variant of lichen planus where a lichenoid reaction can be observed around the acrosyringium [6]. In addition to the conventional lichen planus pathologic findings, perieccrine lymphocytic inflammation was noted with permeation of the lymphocytes into the eccrine coils [7]. It is important to recognize that syringotropism can be a feature of lichen planus as perieccrine inflammation with syringotropic lymphocytes is a common pathologic feature of syringotropic MF and thus may be misdiagnosed as such.

Eccrine involvement in lichen planus has especially been described in palmoplantar lichen planus. Two cases of punctate hyperkeratosis over the palms have been reported that on histopathology exhibited features of lichen planus with eccrine involvement. In addition to the classical findings of lichen planus, a lymphocytic infiltrate around the acrosyringium was noted as well as degeneration of the acrosyringeal basal cell layer [8,9].

The syringotropism observed in LP has been hypothesized to be a result of the extravasation of sweat. The pro-inflammatory cytokines found in the sweat may result in a pro-inflammatory cascade and the recruitment of lymphocytes to the eccrine glands [1,2,10]. In fact, this leakage of sweat has been hypothesized to be one of the early events contributing to the pathogenesis of lichen planus [10].

### 2.2. Graft Versus Host Disease

Graft versus host disease (GVHD) is a multiorgan disease that is commonly associated with hematopoietic stem cell transplantations. The skin is the most commonly affected organ. GVHD exists in both acute and chronic forms. The clinical presentation of GVHD is a vast spectrum that ranges from pruritus, erythema, morbilliform eruptions, to a scleroderma-like picture or even a toxic epidermal necrolysis-like reaction. Histologically, GVHD is characterized by an interface dermatitis with necrotic keratinocytes and satellite cell necrosis [1,2].

**Figure 2:**
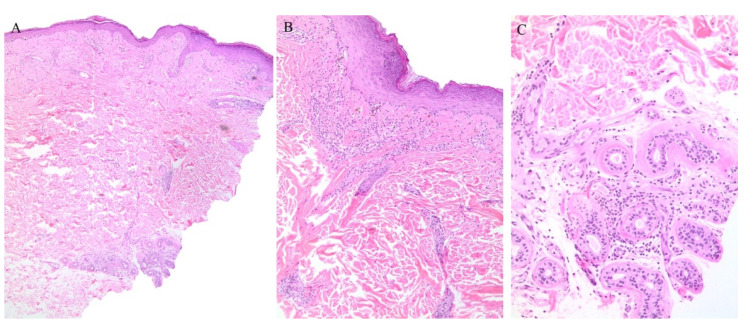
(**A**–**C**) Graft versus host disease with perieccrine inflammation.

Clinically, the involvement of the hair follicle in GVHD can result in a variety of manifestations including non-scarring and scarring alopecias, a follicular rash, and comedonal lesions, among others [11]. Histologically, follicular variants have been described in both acute and chronic forms of GVHD (Figure 2) [12]. Unfortunately, the reported cases of acute follicular GVHD carried a high mortality rate while chronic follicular GVHD seems to confer a more favorable prognosis [13,14]. This involvement of the follicular epithelium has been attributed to inflammation around the bulge region of the hair follicle, likely due to the presence of stem cells in this area [15].

Eccrine gland involvement is also seen in GVHD. In addition to the classical histologic features, a lymphoplasmacytic infiltrate around the eccrine coils is a specific feature of GVHD (Figure 2) [16]. Eccrine squamous syringometaplasia has also been described in biopsy specimens of a patient with cutaneous and hepatic GVHD [17].

### 2.3. Lichen Nitidus

Lichen nitidus (LN) is characterized by an eruption of skin-colored, dome-shaped papules over the body but with a predilection for the extremities and genitalia. The eruption may be asymptomatic or associated with pruritus. On histopathology, the characteristic “ball and claw” appearance is seen. The ball consists of a collection of lymphocytes underlying a slightly atrophic epidermis with basal cell vacuolization. The infiltrate may be band-like or at times granulomatous. This is flanked on either side by dermal papillae, forming the claw [1,2].

**Figure 3:**
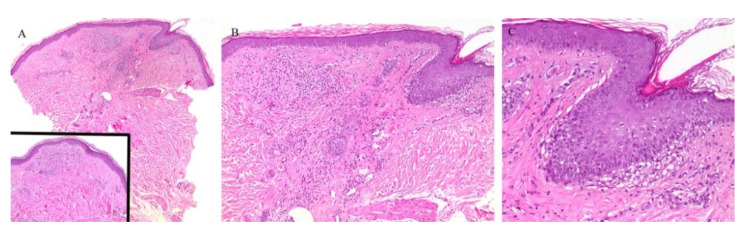
(**A**–**C**) Lichen nitidus with follicular involvement (the inset in Figure A shows a deeper section of the same case with more characteristic lichen nitidus changes).

Appendageal involvement has been described. A perifollicular lymphohistiocytic inflammation has been observed in some cases of LN (Figure 3) [18,19]. In addition, a specific clinical variant of LN has been described whereby the papules had a spinous follicular appearance. On histopathology, in addition to the classical LN findings, a granulomatous infiltrate around hair follicles was noted [20,21,22]. The presence of these perifollicular granulomas led authors to emphasize that this clinical variant may be misread as lichen scrofulosorum [20].

Perieccrine inflammation has also been reported in cases of LN. A lymphocytic eccrine hidradenitis was observed in histopathological specimens of LN, not unlike perieccrine findings seen in lichen striatus [19].

### 2.4. Lichen Sclerosus et Atrophicus

Lichen sclerosus et atrophicus (LSA) is another interface dermatitis that presents with porcelain-white lesions with associated atrophy, most commonly over the anogenital area. There is a strong female predilection. Histopathology reveals an atrophic epidermis, interface changes, and homogenization of collagen in the upper dermis [1,2].

**Figure 4:**
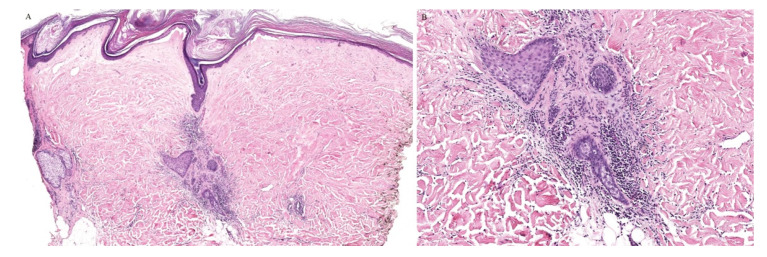
(**A**,**B**) Lichen sclerosus with perifollicular inflammation.

A clinically folliculocentric variant of lichen sclerosus has only been reported twice before in the literature. These cases had extragenital presentations of typical LSA lesions distributed in a perifollicular pattern. In both cases, histopathologic examination did not reveal a follicular infiltrate [23,24]. Nonetheless, an inflammatory infiltrate around the hair follicle can be observed in histopathologic specimens of lichen sclerosus, even in the absence of follicular involvement clinically (Figure 4).

A syringotropic variant has also been described in a patient with hypopigmented atrophic macules on the dorsa of his feet. Histopathology revealed classical pathologic findings of LSA in addition to eccrine duct dilation, lymphocytic inflammation around the eccrine glands, and a band-like lymphocytic infiltrate around the acrosyringium [25].

### 2.5. Lichenoid Drug Eruptions

Cutaneous eruptions in response to a medication are commonly encountered in the field of dermatology. These eruptions vary from papulosquamous, psoriasiform, to lichenoid, among others. Lichenoid drug eruptions may occur with a variety of medications, including antihypertensives, diuretics, antimalarials, and anticonvulsants, among others. These lichenoid drug eruptions may be accompanied by adnexotropism. An eruption of follicular papules that exhibit a lichenoid pathology on biopsy with perifollicular inflammation has also been described in patients who received pegylated liposomal doxorubicin [26,27]. Similarly, a lichenoid eruption with follicular dyskeratosis has been described in a patient receiving anti-tuberculosis therapy [28].

Adnexotropic lichenoid drug eruptions, in particular, have been reported in patients receiving immune checkpoint inhibitors [29]. A lichenoid reaction surrounding the superficial portion of the acrosyringium and the follicular ostia has been reported to occur in up to 45% of cases [30].

In addition, adnexotropic lichenoid drug reactions have also been reported in patients receiving receptor tyrosine kinase inhibitors. A lichen planopilaris-like presentation has been observed with a perifollicular lymphocytic infiltrate being described in these patients [31,32].

## 3. Interface Dermatitides with Microscopic Adnexotropic Features

This group encompasses interface dermatitides in which a peculiar clinical adnexotropic variant has not been reported, but which have been described to exhibit adnexotropic involvement on microscopy.

### 3.1. Connective Tissue Diseases

**Figure 5:**
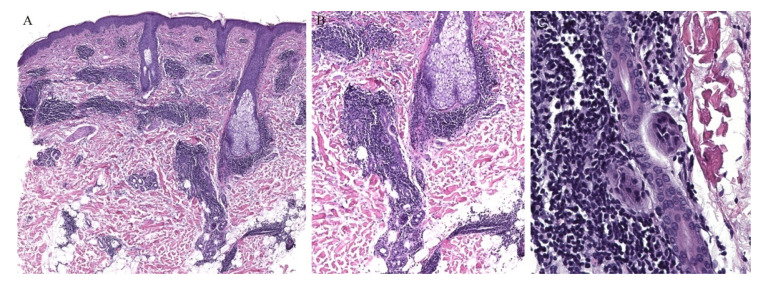
(**A**–**C**) Lupus erythematosus with periadnexal inflammation.

Connective tissue diseases are classically associated with periadnexal inflammation. The classic histopathologic findings of cutaneous lupus erythematosus include an interface dermatitis with basal cell vacuolization as well as a lymphocytic perivascular and periadnexal inflammatory cell infiltrate (Figure 5) [33]. In challenging cases, increased mucin deposition and direct immunofluorescence features may help differentiating connective tissue diseases from mimickers.

### 3.2. Lichen Striatus

**Figure 6:**
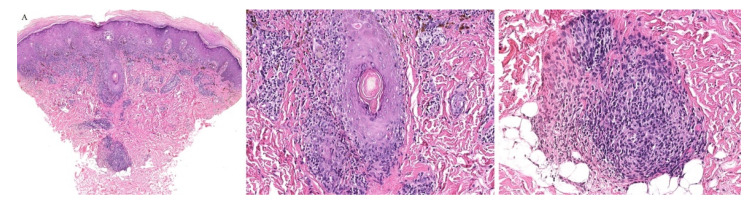
(**A**–**C**) Lichen striatus with perifollicular and perieccrine inflammation.

Lichen striatus (LS) is an uncommon, self-limiting eruption of linear papules that follow the lines of Blaschko. The histopathologic findings are generally non-specific. A lichenoid infiltrate with interface dermatitis can be seen, often with a spongiotic dermatitis as well [1,2]. Adnexal involvement is typical. Perifollicular inflammation has been noted in lichen striatus biopsy specimens [34] (Figure 6). In one study looking at the histologic features of thirty-seven cases of lichen striatus, appendageal involvement, either perifollicular or perieccrine, was observed in 92% of the cases. Many cases often displayed both follicular and sweat gland involvement at the same time. Given the otherwise non-specific findings of lichen striatus, this appendageal involvement prompted the authors to suggest this finding to be part of the diagnostic histopathologic criteria of lichen striatus [35].

The syringotropic feature of LS has also been described in another study. Not only was lymphocytic infiltration of the secretory coil of the eccrine gland noted in all cases studied, but also the presence of eccrine hyperplasia was observed in two cases. Eccrine hyperplasia is a feature of syringotropic mycosis fungoides. Although the clinical presentations of lichen striatus and syringotropic mycosis fungoides are vastly different, their histopathologic features may overlap, and thus awareness of this syringotropic feature of lichen striatus is of utmost importance for dermatopathologists [36].

### 3.3. Pityriasis Lichenoides

Pityriasis lichenoides (PL) encompasses a variety of dermatoses including the acute ulceronecrotic form, pityriasis lichenoides et varioliformis acuta (PLEVA), as well as pityriasis lichenoides chronica (PLC), which presents as scaly papules and plaques.

Histologically, both forms present with similar findings. Parakeratosis, acanthosis, necrotic keratinocytes, and interface changes are all features of PL. A wedge-shaped perivascular lymphocytic infiltrate can also be seen. The histologic findings of PLEVA are usually more severe than those of PLC [1,2].

**Figure 7:**
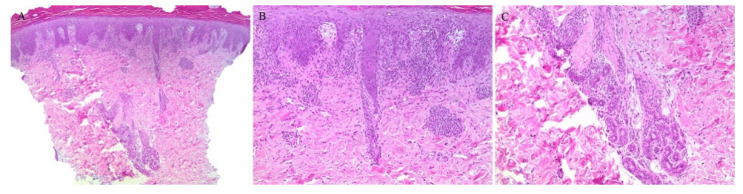
(**A**–**C**) Pityriasis lichenoides et varioliformis acuta with perieccrine inflammation.

**Figure 8:**
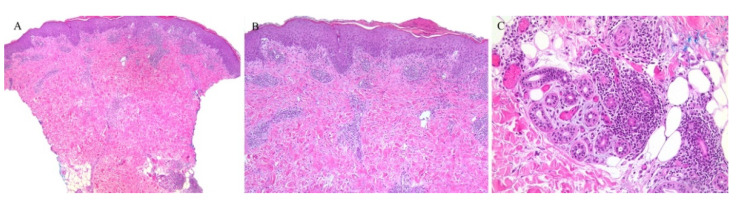
(**A**–**C**) Pityriasis lichenoides chronica with perieccrine involvement.

Although the inflammatory infiltrate of PL is commonly observed around the blood vessels, adnexotropic variants of PL have been described (Figure 7 and Figure 8). In one retrospective review, adnexotropism was observed in 97% of the specimens studied. It was also found that a denser periadnexal inflammation was observed in PLEVA/PLC overlap cases. This pattern of adnexotropism prompted the authors to suggest the descriptive term of a “T-shaped” inflammatory cell infiltrate, rather than a wedge-shaped infiltrate [37]. Syringotropic lymphocytes has been reported in one case of acral pityriasis lichenoides [38].

### 3.4. Keratosis Lichenoides Chronica

Keratosis lichenoides chronica (KLC) is another lichenoid dermatosis characterized by erythematous hyperkeratotic lichenoid papules often arranged in a linear distribution. Histologically, KLC exhibits a lichenoid eruption with hyperkeratosis, parakeratosis, and acanthosis. The dermis displays a band-like infiltrate with lymphocytes and histiocytes [1,2]. This infiltrate has also been reported to surround the acrosyringia of the eccrine units [39]. Eccrine squamous syringometaplasia has also been observed in KLC cases [40].

### 3.5. Lichen Aureus

**Figure 9:**
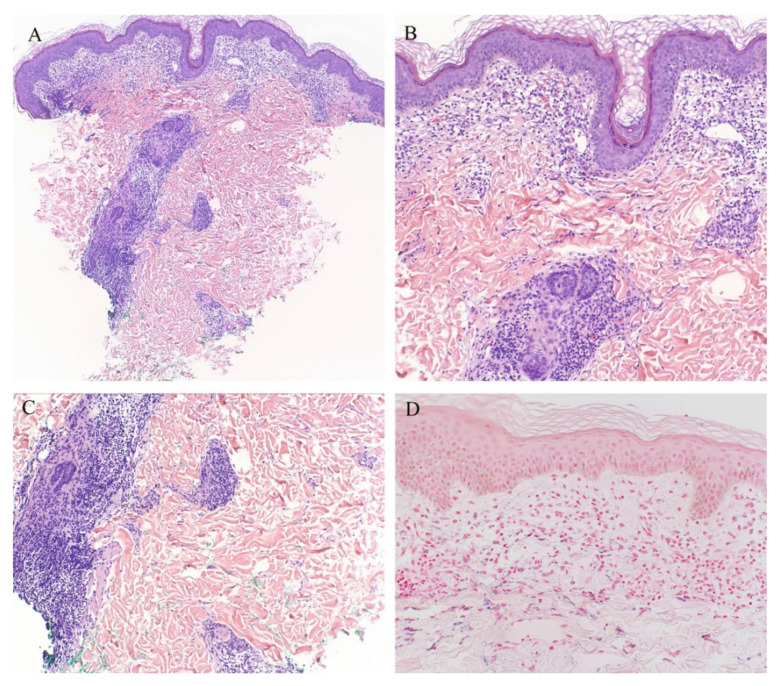
(**A**–**C**) Lichen aureus with periadnexal inflammation; (**D**) Iron stain.

Lichen aureus is a subtype of the pigmented purpuric dermatoses which, clinically, usually presents as solitary confluent golden lichenoid papules or macules commonly on the legs. On histopathology, a band-like lymphohistiocytic infiltrate can be seen in the upper dermis without basal cell vacuolization. Red cell extravasation may also be seen [41,42]. Lichen aureus may also demonstrate periadnexal inflammation (Figure 9) [41,42]. One study noted periadnexal inflammation in 48% of cases, being the second most common finding after hemosiderin deposition [43]. The classical histopathologic features of lichen aureus including periadnexal inflammation and, at times, perineural inflammation can be confused with lichen striatus, which can exhibit similar findings [42].

## 4. Mimickers

### 4.1. Mycosis Fungoides

Mycosis fungoides (MF) is the most common form of primary cutaneous lymphoma. Classically, MF is characterized on histopathology by the presence of infiltrating atypical lymphocytes, namely epidermotropism, into the epidermis [44]. Different variants of the disease are recognized including the adnexotropic variants, namely the folliculotropic and the syringotropic MF. These are important entities that must be excluded when encountering periadnexal interface dermatitides.

**Figure 10:**
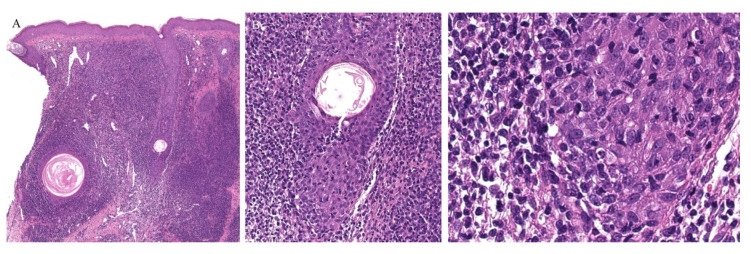
(**A**–**C**) Histopathology of follicular mycosis fungoides.

Follicular MF is a variant of MF whereby the atypical lymphocytes infiltrate the hair follicle (Figure 10). Clinically, this condition is mainly seen over the head and neck areas and may be associated with alopecia.

Histopathologically, follicular MF is usually characterized by a mildly atypical, variably pleomorphic lymphocytic infiltrate with perifollicular accentuation with associated cysts in up to 36% of cases. The epidermis is commonly spared. Mucin deposits in the follicular epithelium (follicular mucinosis) characterizes FMF and has been described in around 75% of cases. In addition, concomitant perieccrine infiltrates have been reported in 4% to 33% of cases. Distinguishing between follicular MF and follicular mucinosis may be challenging, however necessary, as the two entities vary in outcomes and prognosis. In the case of follicular mucinosis, follicular involvement may be observed in the form of mucinous degeneration of the hair follicle [45]. Follicular mucinosis in the absence of mycosis fungoides confers a benign course. However, in follicular MF, intrafollicular mucin deposition secondary to the lymphocytic infiltrate of the follicular epithelium can be observed [46].

**Figure 11:**
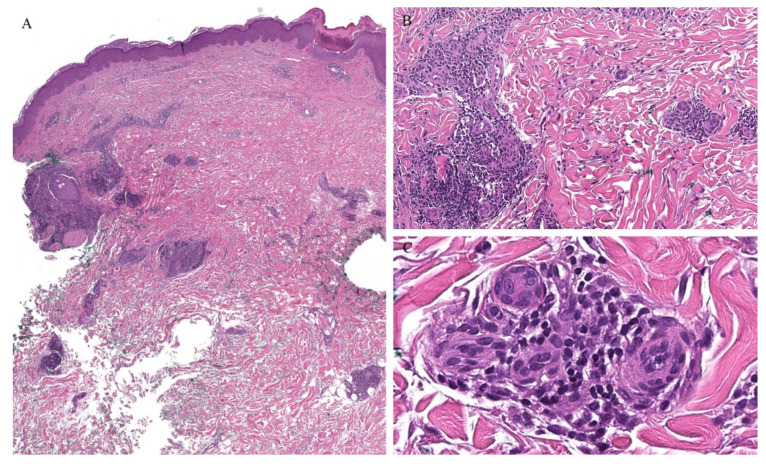
(**A**–**C**) Histopathology of syringotropic mycosis fungoides.

Syringotropic MF has also been described with marked eccrine gland and duct epitheliotropism (Figure 11). Clinically, this may appear as a solitary lesion or multiple localized lesions. Alopecia has also been described in syringotropic MF. In addition, syringometaplasia has also been reported in a few cases [47].

Involvement of the hair follicle and eccrine glands have been reported concomitantly within the same biopsy specimens. In one study of patients with folliculotropic MF, eccrine involvement was reported in up to 56% of the cases [46]. In addition, two cases of concomitant folliculotropic and syringotropic MF without any epidermal involvement have been described, illustrating a major pitfall in recognizing MF without the classical features of epidermotropism [48].

In addition to these microscopic features, immunohistochemistry and molecular testing can help in differentiating adnexotropic MF variants from interface dermatitides with adnexotropic features. In MF, immunohistochemistry typically shows expression of pan-T-cell markers with a marked shift in CD4/CD8 ratio in favor of CD4 and possible antigen loss. Monoclonal TCR rearrangement can usually be demonstrated in the majority of cases.

### 4.2. Perniosis

Perniosis, or chilblains, is a dermatosis that presents with inflammatory skin lesions over acral sites, often on exposure to the cold. Basal cell vacuolization, necrotic keratinocytes, and a lymphocytic infiltrate are observed on histopathology. Although the infiltrate is typically perivascular, a perieccrine inflammatory cell infiltrate has been described in cases of both idiopathic and autoimmune perniosis. However, perieccrine inflammation was a finding more commonly seen in idiopathic cases [49,50] with one study reporting this finding in 100% of idiopathic perniosis cases reviewed [51].

Chilblain-like changes have been described during the coronavirus disease 19 (COVID-19) pandemic. This finding has been increasingly reported in younger patients. Basal cell vacuolization and necrotic keratinocytes have been reported in biopsy specimens of these patients. A lymphocytic infiltrate that is mainly perivascular has also been described. Interestingly, a perieccrine lymphocytic inflammatory cell infiltrate has been demonstrated in 30% [52] to 47% of cases [53].

## 5. Conclusions

The interface dermatitides are characterized by the histopathological features of basal cell vacuolization and apoptotic keratinocytes. These entities may exhibit distinct adnexotropic features on histopathology. Infiltration of the inflammatory cells around the hair follicle and the eccrine gland, although not always observed, should be recognized as potential features of these conditions. These changes on histopathology may or may not be accompanied by an adnexotropic clinical counterpart. Some conditions such as lichen planus and lichenoid drug eruptions may appear clinically as perifollicular as well as demonstrate a folliculotropic infiltrate on histopathology. However, in some conditions, clinical adnexal involvement may not be appreciated. 

Understanding that these conditions may show follicular and eccrine inflammation may aid dermatopathologists in recognizing the diagnosis and in avoiding the misinterpretation of such adnexotropism as a strict feature of mycosis fungoides or connective tissue diseases.
